# A survey of nutritional education within the Holiday Activities and Food programme across England

**DOI:** 10.3389/fpubh.2024.1425468

**Published:** 2024-09-04

**Authors:** Emily K. Round, Paul B. Stretesky, Margaret Anne Defeyter

**Affiliations:** ^1^Healthy Living Lab, Northumbria University, Newcastle upon Tyne, United Kingdom; ^2^School of Social and Political Sciences, University of Lincoln, Lincoln, United Kingdom

**Keywords:** nutritional education, holiday provision, holiday clubs, food education, food literacy, food insecurity, school policy, HAF

## Abstract

**Background:**

Nutritional education is a mandatory component of the Holiday Activities and Food (HAF) programme in England, yet there is a paucity of literature exploring how this component is delivered. The aim of this study was therefore to explore the delivery, content, dose and perceived impacts of nutritional education, at the HAF club level, across England.

**Methods:**

A self-completion, cross-sectional online survey design was adopted. Non-probability purposive sampling was used to collect data from HAF club leads (*n* = 147) from across England. Data were analysed using descriptive statistics and frequencies.

**Findings:**

Face-to-face nutritional education was the most common mode of delivery, with sessions mostly comprising of discussing food and nutrition. However, whilst the majority of clubs delivered the required number of nutritional education sessions per week, according to the Department for Education’s guidelines, the time spent delivering individual nutritional education activities may not be sufficient to drive change in related skills and behaviours. Moreover, many clubs did not adopt a whole-family approach, and some did not deliver any nutritional education activities at all, which club leads attributed to a lack of material resources and ambiguity in the national HAF guidance.

**Conclusion:**

Nutritional education is delivered in a variety of ways across HAF clubs, based upon available local assets, resources and venues. Policy and practice recommendations include increased HAF funding to support clubs that lack material resources, national training in nutritional education, and evidence-informed guidance and practice.

## 1 Introduction

The State of School Feeding Worldwide report published by the World Food Programme (1) estimates that across the world, approximately 418 million children benefit from a school feeding programme, often combined with interventions to improve health and wellbeing (2). Indeed, school feeding programmes are often multi-faceted, aiming to reduce hunger, whilst delivering health-related interventions such as nutritional education and de-worming which can, in-turn, incentivise families to attend school (3). For example, in low-middle income Ghana, children attending beneficiary schools receive free meals; this programme attempts to reduce malnutrition and hunger, whilst increasing school enrolment, retention and household income; children involved in this programme have been found to improve in both literacy and numeracy (4).

In England, children from families in receipt of an annual household income of less than £7,400 per year are typically eligible to receive a means-tested free school meal (FSM) each day during school term-time (5). Problematically however, when school is not in session, FSM provision is not usually available. Combined with the cost of childcare and activities, many families consequently face increased financial pressures which can result in increased levels of food insecurity and social isolation during these periods; although families who are not eligible for FSM often face similar issues (6). To overcome such challenges, school holiday clubs have grown in prevalence over recent years, where activities and food are provided to children across the school holidays (7, 8). Alongside significant reductions in food insecurity, holiday clubs afford families with opportunities for socialisation, participating in enriching activities and learning new skills (9–11) but challenges in sustaining provision often relate to limited funding (12).

In 2021, the Holiday Activities and Food (HAF) programme was funded at £220M *per annum* by the Department for Education (DfE) for three financial years. HAF provides school holiday provision to children who are typically in receipt of means-tested FSM across all upper-tier local authorities in England. However, local authorities can utilise up to 15% of their funding allocation to allow children to attend who are not in receipt of means-tested FSM, but may benefit from attendance (13). Funding for HAF is provided by the DfE to all upper-tier local authorities in England. The local authorities then advertise the HAF programme and invite organisations across the statutory, private, community and voluntary sectors to submit applications to be awarded HAF funding. Each local authority then decides which organisations are funded to deliver the HAF programme. The individuals who lead the delivery of HAF within each of these organisations are known as ‘club leads’.

The HAF programme builds upon research regarding holiday clubs (7, 8, 14) and differs from FSM parcel schemes instigated during lockdown which focused solely on meals or monetary support (15). For the HAF programme, the DfE expect local authorities to deliver the equivalent of 6 weeks’ total holiday provision across the Easter, Summer and Christmas school holiday periods. Indeed, over the Easter and Christmas periods, eligible children should be offered at least 4 days’ provision for at least 4 h per day, whilst during the six-week school summer holidays, clubs are expected to offer at least 4 weeks’ provision, covering a minimum of 16 days with 4 h of delivery per day; delivering food that complies with School Food Standards and a range of enriching activities (13). HAF is of significant reach, with the most recent figures indicating that approximately 750,000 children attended a HAF club during summer 2021 (16) and a growing body of literature demonstrates the importance of the HAF programme for children and families. Indeed, alongside attenuating household food insecurity (17), improving dietary intake (11, 18) and increasing levels of physical activity (10), HAF affords children and families enrichment and socialisation opportunities which they otherwise may not experience (6, 7, 19).

Whilst the DfE encourage an assets-based and place-based approach to local programme delivery, a quality standards framework is disseminated to all providers which stipulates mandatory components that clubs must deliver; alongside physical activities, enrichment opportunities, adhering to School Food Standards and following safeguarding protocols, nutritional education must be delivered to those who attend (13). This inclusion is likely related to published literature demonstrating the beneficial impacts of nutritional education within non-HAF contexts. For instance, both community-based and school-based sessions, often including practical food-related experiences, have been associated with improvements in food literacy, nutritional knowledge, cooking skills and confidence, as well as a positive shift in dietary intake (20–22). Furthermore, food and nutritional education is a compulsory component of the national curriculum, and academic research has shown the subsequent benefits of school-based food education such as improved food awareness, cooking skills, self-esteem and willingness to try new foods (23, 24).

Despite nutritional education being a mandatory component of the DfE HAF guidance however, there is a paucity of published literature which investigates how nutritional education is delivered in HAF. Indeed, broad HAF evaluations have only briefly considered nutritional education (16, 25), and only one recent academic study has explored the implementation and delivery of HAF nutritional education through semi-structured interviews with HAF leads at local authority and co-ordinator level (26). The findings of Round et al. (26) informed the design and method of the current study which aimed to explore the delivery, content, dose and perceived impacts of nutritional education, at the HAF club level, across England.

## 2 Materials and methods

### 2.1 Design and participants

This study adopted a self-completion, cross-sectional online survey design and used non-probability purposive sampling to collect data from HAF club leads across England (i.e., the individuals who lead the delivery of HAF within each HAF funded organisation). As the DfE do not share information regarding HAF clubs, there was no national database to inform a sampling framework; hence, the survey link was disseminated via the HAF Alliance (27) and the All Party Parliamentary Group (APPG) on School Food, to all upper-tier local authorities in England. HAF leads within each local authority were asked to subsequently disseminate the link to each of their HAF clubs for the club lead to complete. When participants accessed and consented to participate in the survey, the online system automatically generated each individual a participant number. However, participants were removed from the database if they did not subsequently answer any survey questions. In order to ensure robust data collection and storage processes, we used the automatically generated participant numbers for data analysis. Responses were removed if they did not deliver provision during the school holidays (*n* = 3), were located in Scotland or Northern Ireland (*n* = 2) or they had closed their browser and restarted the survey (*n* = 3), which resulted in a final sample of 147 HAF club leads. Demographic information was provided by 111 participants, who identified as male (*n =* 34), female (*n =* 71), other (*n =* 1) or preferred not to say (*n =* 5) and were aged between 22 and 76 (Mean age = 43.2 years, SD = 13.8). Participants reported that they were Asian (*n =* 2), Black (*n =* 4), Mixed (*n =* 1), White (*n =* 96), other (*n =* 2) or preferred not to say (*n =* 6).

### 2.2 Online survey

The online survey was hosted on Qualtrics and was accessible on a computer, laptop, tablet or smartphone. The survey included 17 main questions, with 10 follow-on questions. Whilst closed questions were primarily included, four open questions allowed participants to explain their responses: (1) “Are there any additional facilities that would help you to deliver nutritional education? If so, please state/explain these using the text box below,” (2) “What would help improve staff and volunteers’ skills and experience?,” (3) “Please state how satisfied you are with the nutritional education that your club delivers and please explain why you chose this level of satisfaction” and, (4) “How much does your club rely on the advice and guidance from the DfE for nutritional education? Please explain why you think this.” All participants were given the opportunity to win one of four £100 e-vouchers by submitting their email address at the end of the survey, as a thank you for participation. The survey was active between 24th May and 24th November 2022.

### 2.3 Procedure

Ethical approval was granted from the Faculty of Health and Life Sciences Ethics Committee at Northumbria University (reference number: 45913). A draft survey was piloted by one HAF lead and three experienced researchers to eliminate system faults and enhance clarity of survey questions. The survey link was emailed to the HAF Alliance and APPG on School Food, who disseminated the link to all upper-tier local authorities in England. Local authority HAF leads were then asked to disseminate the survey to each of their HAF clubs for the respective club lead to complete.

On the first page of the survey, an information sheet and consent form were presented. If participants did not consent, they were automatically referred to the end of the survey. Participants were asked to provide a memorable codeword for anonymity and to use if they wished to withdraw their anonymous data. The 17 main questions, and 10 follow on questions, regarded the delivery, content, dose (how often it was delivered), users and perceived impacts of HAF nutritional education. Participants were additionally asked to provide personal demographic information (e.g., age, gender, and ethnicity), as well as the postcode of their club and the local authority from which HAF funding was acquired. In total, the survey took approximately 20 minutes to complete. A debrief sheet was presented on the last page of the survey, which outlined the purpose of the study, explained participants’ right to withdraw within a given timeframe, and provided researcher contact information.

### 2.4 Data analysis

Data were removed where HAF club leads did not deliver provision during the school holidays (*n* = 3), were located in Scotland or Northern Ireland instead of England (*n* = 2) or it was clear that they had closed their browser and restarted the survey (*n* = 3). Postcode data were additionally checked to verify that participants did not complete the survey twice.

Responses from closed questions were coded and analysed using SPSS version 28 and are presented as descriptive statistics and frequency tables. For questions where participants stated the number of hours they delivered activities, data were re-coded into seven categories from ‘0 hours’ to ‘11+ hours,’ to identify patterns in the data more easily. Moreover, combined totals were computed by collating the hours per week club leads stated they spent organising and/or delivering each type of nutritional education overall (e.g., face-to-face, take-home, online, other), as well as for the overall amount of time per week they spent delivering nutritional education activities (e.g., discussing food and nutrition, practical food education, online information). As the number of incomplete responses increased towards the end of the survey, suggesting cumulative attrition due to fatigue rather than question avoidance, unanswered questions were defined as ‘missing’ and excluded from analysis.

For open ended questions, data were coded and collated to form common categories relating to club leads’ experiences of HAF nutritional education. In addition, where participants stated they delivered ‘other’ types of nutritional education, responses were grouped.

## 3 Results

For organisational purposes, the survey questions were categorised into: (1) venues and facilities available and used to deliver nutritional education, (2) nutritional education delivery, (3) perceived impacts of nutritional education, and (4) guidance, skills and experience required to deliver nutritional education. For qualitative responses, quotes are stated alongside participant numbers and a code to indicate which venue they operated from (i.e., community centre = CC, school = S, leisure centre = LC, faith-based = F, park = P, other = O, mixed = M).

### 3.1 Venues and facilities available and used to deliver nutritional education

HAF club leads indicated the type of venue(s) from which their club operated. If they operated from more than one venue, responses were categorised as ‘mixed’.

As show in Table 1, 38.8% of HAF clubs operated from schools. However, collapsing data across all other venues (i.e., community centres, leisure centres, faith-based organisations, parks/playing fields, other) demonstrated that most clubs operated from community settings (52.4%). In addition, 8.8% of clubs operated from multiple (‘mixed’) venues; sometimes clubs do not operate from one venue only (i.e., operate from a community centre 3 days per week and a school 2 days per week). The ‘other’ venues that clubs operated from included youth clubs, theatres, sports venues, museums, arts centres, adventure playgrounds and one club even used an adapted double-decker bus.

**Table 1:** Frequency table showing responses regarding the venue from which HAF holiday clubs operate (*n =* 147).

	*N*	%
Community centre	13	8.84
School	57	38.78
Leisure centre	10	6.80
Faith-based organisation	3	2.04
Park/playing field	3	2.04
Other	48	32.65
Mixed – more than one of the above venues	13	8.84
Total	147	100

Participants were additionally asked what kitchen facilities were available at their HAF club to support the delivery of nutritional education. Data were collapsed across the responses of ‘fully fitted kitchens that children can access and use,’ ‘partial fitted kitchens that children can access and use,’ ‘portable cooking facilities,’ and a ‘mixture of cooking facilities.’ 37.2% of clubs did not have access to kitchen facilities (Table 2).

**Table 2:** Frequency table displaying the kitchen facilities available at HAF clubs to deliver nutritional education (*n =* 145).

	*N*	%
Fully fitted kitchen that children can access and use	36	24.83
Partial fitted kitchen that children can access and use	11	7.59
Portable cooking facilities	17	11.72
Mixture of cooking facilities (fully fitted kitchen, partial fitted kitchen and/or portable cooking equipment)	27	18.62
No kitchen facilities	54	37.24
Total	145	100

Similarly, club leads were asked about the available space at their club to deliver nutritional education. Data were collapsed across responses of ‘indoor space only,’ ‘outdoor space only,’ and ‘mixed types of space’ (indoor, outdoor, gardening/growing spaces). Most HAF clubs (63%) had access to mixed spaces to deliver nutritional education, with 15% having access to indoor space only and less than 5% having access to outdoor space only. Nearly 17% of clubs had no suitable spaces available to deliver nutritional education.

In addition, 9% of HAF clubs had access to alternative facilities related to nutritional education delivery, which included a servery, campfires, outdoor kitchens, poly-tunnels, and sensory rooms for SEND children. Most club leads were ‘very satisfied’ or ‘satisfied’ with the facilities available (79.6%), with only 3.5% reporting that they were ‘dissatisfied’ or ‘very dissatisfied’. Regardless of their reported level of satisfaction, participants were asked what facilities would improve their nutritional education offer. A total of 54 participants responded to this question, with responses categorised into (1) additional kitchen facilities and equipment, (2) additional educational resources, and (3) additional skilled staff.

Suggestions regarding additional kitchen facilities and equipment were made by 29 club leads, which highlighted that “access to simple cooking equipment” (P152-M) and “a kitchen big enough for all the children to cook in” (P6-S) would improve their nutritional education offer. Where clubs operated from schools, club leads acknowledged that “school facilities are in use to cook for the children but not available for use by club staff” (P211-S) so further “access to a school kitchen would help” (P221-O). Moreover, club leads highlighted that “finances to install cookers and better kitchen facilities” (P202-O) and “catering equipment to demonstrate and involve children more” (P82-CC) would be beneficial, alongside access to facilities and amenities linked to health, safety and hygiene, such as “running hot water for washing hands and veg, and electricity for a fridge, hob or oven” (P216-O), and a “freezer to store large shopping needs” (P82-CC).

In addition, 18 club leads noted that access to additional educational resources, including “purpose-built learning areas” (P208-O), as well as “classrooms and PowerPoint provisions to deliver healthy eating and choices workshops” (P71-O) would improve their delivery. Club leads believed that outdoor areas and gardening resources such as “vegetable planting beds and fruit hanging baskets” (P171-CC) would “enable us to garden and grow across the year and then use the produce to prepare healthy wraps and smoothies” (P171-CC). Moreover, additional educational resources such as “a scheme of work booklet” (P21-S) or “3D models of the human body to show children what the inside of our body looks like and how food moves through it” (P127-S) were recognised as resources that leads would like access to.

Moreover, seven club leads thought outsourcing to, or recruitment of, skilled food-related professionals, who are trained in food and nutrition, would improve their nutritional education offer, such as a “chef on-site” (P105-O) and “a public health team to support, as well as a tutor trained in nutrition advice” (P2-M). One participant explained that they had already implemented this and believed it had improved their delivery: “we can provide a basic level of nutritional education including access to books, online content, shopping lists and worksheets. However, to improve our offer we are now working with a partner” (P177-O).

Facilities and resources were also highlighted by 44 club leads as factors impacting their satisfaction with their nutritional education offer. For instance, 10 club leads highlighted that having staff who are skilled and experienced in delivering cooking sessions at their club “gives [families] the opportunity to try foods, get hands-on in preparation and eat their finished meal” (P10-P). Furthermore, three club leads acknowledged that access to growing space allowed for high quality, practical nutritional education regarding food origins and contents at their club: “children can see and learn about produce growing in the garden, pick and sample and cook with them. We talk about what is healthy and why” (P219-O). Indeed, clubs that had the capacity to grow fruits and vegetables had 5.8 times the odds of engaging in practical food education activities [*χ*^2^ = (1, *N* = 120) = 23.9, *p* < 0.05] and 10.3 times the odds of engaging in sensory food education [*χ*^2^ = (1, *N* = 120) = 14.4, *p* < 0.05]. However, others acknowledged that a lack of facilities and resources hindered the quality of nutritional education they could provide. For example, one club lead, whose club operated from a sports facility, noted that they had “very limited facilities to be able to offer satisfactory nutritional education to participants” (P179-O) and another lead, whose HAF club operated from multiple venues, said they could “do more preparation of food and food tasting if we had better access to kitchen facilities” (P37-M). Even where HAF clubs operated from schools, kitchens were often inaccessible for nutritional education during the school holidays, which limited the provision that could be delivered: “schools do not grant permission from non-catering staff to use the kitchens as we are a charity using their facilities, so they do not want to risk misuse or damage. It is difficult to provide even basic cooking or sampling without access to fridges and cooking equipment which is why we do a lot of theory rather than practical” (P211-S).

When asked how staff/volunteers’ skills and experience could be improved, four club leads highlighted that access to further resources and facilities “for children to access safely” (P82-CC), including “more facilities for children to make food” (P5-S) would allow staff to deliver high quality provision. Participants highlighted that “not all venues have access to these facilities” (P20-S). For instance, one of the respondents noted that “some venues, such as leisure centres, do not have kitchen facilities so food activities are more difficult to run” (P37-M) and that this “can limit what is achievable” (P37-M) for nutritional education, suggesting variation across clubs in terms of accessibility of facilities which may impact the type and quality of the nutritional education offer. Moreover, 18 club leads noted additional resources which include examples of nutritional information, such as “literature to send home” (P31-S), “recipe cards which staff could use and then share with parents” (P6-S) and “localised information that we can add to signposting, e.g., foodbanks, how to order groceries online” (P16-S), would improve staff/volunteers’ skills and experience regarding appropriate, high-quality content. One club lead believed “access to information in alternate languages” (P16-S) would be beneficial for children and families with English as an additional language.

### 3.2 Delivery of nutritional education

Participants were asked how many hours per week their HAF club spent organising and/or delivering nutritional education through face-to-face, take-home (e.g., any form of nutritional education at home provided by the HAF club; leaflets, recipes), online (e.g., websites, videos, and online teaching resources) or ‘other’ methods. Face-to-face nutritional education was the most commonly used mode of delivery (77.7%), In addition, 8.5% of clubs organised and/or delivered ‘other’ types of nutritional education, which included food shopping, discussing healthy options during mealtimes, linking to relevant support at the venue where HAF was delivered, and using smartphone applications.

Whilst identified as the most common mode of delivery however, the majority of clubs did not spend more than 2 h per week organising and/or delivering face-to-face nutritional education. For instance, 34.6% of clubs only delivered face-to-face nutritional education for 1–2 h per week, and a further 22.3% of clubs did not organise and/or deliver any face-to-face nutritional education at all. Likewise, 36.9% of clubs only spent 1–2 h per week organising and/or delivering take-home nutritional education, with an additional 47.7% of clubs not organising and/or delivering any take-home content. Moreover, online content was organised and delivered by 16.9% of clubs for only 1–2 h per week, with a further 74.6% of clubs not organising and/or delivering any online nutritional education at all.

When data were collapsed across all modes of delivery (i.e., collating the hours they spent in total on nutritional education organisation and delivery per week), over half of clubs (60.8%) spent more than 2 h per week organising and/or delivering nutritional education. This suggests that clubs often use multiple modes of delivery for nutritional education. It is, however, concerning that 18.5% of HAF clubs did not spend any time organising and/or delivering any mode of nutritional education at all.

Club leads were asked how many hours per week they delivered specific nutritional education activities to children. The most common activity was discussing food and nutrition, delivered by 70% of HAF clubs. However, most clubs did not spend more than 2 h per week delivering this activity. Indeed, 40% of clubs only delivered this activity for 1–2 h per week, with an additional 30% of clubs not delivering this activity at all. Likewise, 16.7% of clubs only delivered practical food activities (e.g., food preparation, assembling, cooking) for 1–2 h per week, with a further 31.7% of clubs not delivering any practical food activities to children. Furthermore, 33.3% of clubs only delivered sensory food education for 1–2 h per week, with an additional 45.8% of clubs not delivering this activity to children at all. For all other activities (e.g., growing/harvesting, online information), a greater percentage of clubs did not deliver each respective activity to children than the percentage of clubs that did (Table 3).

**Table 3:** Frequency table displaying hours per week spent delivering separate nutritional education activities to children at HAF clubs (*n =* 120).

	*N*	%
Practical food activities (e.g., food preparation, assembling, cooking)		
0 hours	38	31.67
1–2 hours	20	16.67
3–4 hours	19	15.83
5–6 hours	13	10.83
7–8 hours	6	5.00
9–10 hours	5	4.17
11+ hours	19	15.83
Total	120	100
Growing fruit/vegetables/herbs		
0 hours	82	68.33
1–2 hours	29	24.17
3–4 hours	5	4.17
5–6 hours	0	0.00
7–8 hours	1	0.83
9–10 hours	1	0.83
11+ Hours	2	1.67
Total	120	100
Sensory food education		
0 hours	55	45.83
1–2 hours	40	33.33
3–4 hours	12	10.00
5–6 hours	8	6.67
7–8 hours	1	0.83
9–10 hours	2	1.67
11+ hours	2	1.67
Total	120	100
Discussing food and nutrition		
0 hours	36	30.00
1–2 hours	48	40.00
3–4 hours	14	11.67
5–6 hours	16	13.33
7–8 hours	1	0.83
9–10 hours	2	1.67
11+ hours	3	2.50
Total	120	100
Online nutrition information (e.g., websites, videos, online school teaching resources)		
0 hours	81	67.50
1–2 hours	29	24.17
3–4 hours	7	5.83
5–6 hours	2	1.67
7–8 hours	1	0.83
9–10 hours	0	0.00
11+ hours	0	0.00
Total	120	100
Other		
0 hours	105	87.50
1–2 hours	8	6.67
3–4 hours	2	1.67
5–6 hours	4	3.33
7–8 hours	0	0.00
9–10 hours	0	0.00
11+ hours	1	0.83
Total	120	100
Overall		
0 hours	18	15.00
1–2 hours	13	10.83
3–4 hours	9	7.50
5–6 hours	19	15.83
7–8 hours	8	6.67
9–10 hours	15	12.50
11+ hours	38	31.67
Total	120	100

However, when the hours clubs spent in total on all nutritional education activities for children per week were collated, the majority of HAF clubs (72.4%) delivered nutritional education activities for more than 2 h per week. This suggests that clubs deliver nutritional education to children through a range of activities. 15% of clubs did not deliver nutritional education to children at all (Table 3).

Likewise, club leads were asked about the number of hours per week they delivered nutritional education activities for parents/carers. Once again, discussing food and nutrition was the most commonly delivered activity for parents/carers (32.5%), however only 20% of clubs delivered this activity for 1–2 h per week, and 67.5% of clubs did not deliver this activity at all. For parents/carers, a greater percentage of clubs did not deliver each respective activity than the percentage of clubs that did. Furthermore, collating the total number of overall hours clubs spent on nutritional education activities showed that most HAF clubs (54.2%) did not deliver any nutritional education activities for parents/carers (Table 4).

**Table 4:** Frequency table displaying hours per week spent delivering separate nutritional education activities to parents/carers at HAF clubs (*n =* 120).

	*N*	%
Practical food activities (e.g., food preparation, assembling, cooking)		
0 hours	91	75.83
1–2 hours	13	10.83
3–4 hours	7	5.83
5–6 hours	1	0.83
7–8 hours	0	0.00
9–10 hours	0	0.00
11+ hours	8	6.67
Total	120	100
Growing fruit/vegetables/herbs		
0 hours	102	85.00
1–2 hours	11	9.17
3–4 hours	3	2.50
5–6 hours	0	0.00
7–8 hours	2	1.67
9–10 hours	1	0.83
11+ hours	1	0.83
Total	120	100
Sensory food education		
0 hours	96	80.00
1–2 hours	13	10.82
3–4 hours	5	4.17
5–6 hours	2	1.67
7–8 hours	2	1.67
9–10 hours	2	1.67
11+ hours	0	0.00
Total	120	100
Discussing food and nutrition		
0 hours	81	67.50
1–2 hours	24	20.00
3–4 hours	7	5.83
5–6 hours	5	4.17
7–8 hours	1	0.83
9–10 hours	1	0.83
11+ hours	1	0.83
Total	120	100
Online nutrition information		
0 hours	89	74.17
1–2 hours	24	20.00
3–4 hours	5	4.17
5–6 hours	1	0.83
7–8 hours	1	0.83
9–10 hours	0	0.00
11+ hours	0	0.00
Total	120	100
Budgeting information		
0 hours	86	71.66
1–2 hours	27	22.50
3–4 hours	5	4.17
5–6 hours	0	0.00
7–8 hours	2	1.67
9–10 hours	0	0.00
11+ hours	0	0.00
Total	120	100
Other		
0 hours	111	92.50
1–2 hours	8	6.67
3–4 hours	1	0.83
5–6 hours	0	0.00
7–8 hours	0	0.00
9–10 hours	0	0.00
11+ hours	0	0.00
Total	120	100
Overall		
0 hours	65	54.17
1–2 hours	14	11.67
3–4 hours	8	6.67
5–6 hours	13	10.83
7–8 hours	7	5.83
9–10 hours	3	2.50
11+ hours	10	8.33
Total	120	100

Participants were also asked how satisfied they were with the nutritional education their HAF club delivered. Collapsing data across ‘very dissatisfied’ and ‘dissatisfied’ found that 13% of club leads were unhappy with their offer, although collapsing data across responses of ‘satisfied’ and ‘very satisfied’ found that 60.9% of club leads were happy with the nutritional education their club delivered. A total of 26.1% of HAF club leads were neither dissatisfied nor satisfied with the nutritional education that their club delivered. To enhance clarity surrounding these responses, HAF club leads were asked why they chose this level of satisfaction. Responses (*n =* 96) were categorised into (1) facilities and resources, (2) parental engagement, (3) logistics, and (4) nutritional education was not a priority.

Facilities and resources were highlighted by 44 club leads as factors impacting their satisfaction with their nutritional education offer, and these data are discussed in sub-section 3.1.

In addition, 16 club leads explained that parental engagement with nutritional education influenced their levels of satisfaction. Of these 16 club leads, eight were satisfied with the nutritional education their club delivered, as “the people who lead workshops are highly qualified and the children and parents get a lot out of it” (P93-S) and their clubs offered “a diverse range of nutritional education from family engagement, where families prepare, cook and eat the food together, to children learning simple cooking skills” (P101-M). However, the remaining eight club leads were not satisfied with their club’s delivery; they “would like to provide more information to the parents” (P209-O) as they believed “if parents learned alongside their children, this would have more of an impact – if parents/carers and children could prepare, cook and eat a meal together during the holiday club for instance” (P171-CC). Club leads noted that they were often “bombarded with questions from parents so there is a need [to include them]” (P221-O) but “found that they are reluctant to get involved, even when food has been sent home for them to prepare” (P134-O). Club leads felt levels of satisfaction would likely improve if parents were more involved in nutritional education sessions, particularly as the children “are not the ones choosing or paying for food at home” (P220-O).

Likewise, the logistics surrounding nutritional education delivery also influenced club leads’ (*n =* 18) reported levels of satisfaction. For instance, one club lead explained that as they mainly delivered sports provision, it was “logistically difficult to provide a nutritional element into the programme” (P45-M). Another provider worked in a detached setting with teenagers and noted that they were “limited by the lack of resources and access, as well as the attention timeframe with teenagers whilst on the street” (P4-P) and often “have to work quickly and smartly in order to get the messages and opportunities across, before they leave for other activities” (P4-P). Capacity and time constraints were also influential to the nutritional education offer, and subsequently impacted club leads’ satisfaction. For instance, one club lead said they “do not have capacity to deliver nutritional education when preparing and cooking food for over 100 young people” (P229-O) whilst four others were aware of how to improve their nutritional education delivery, but noted that implementing such changes proved difficult due to time-related barriers: “there is not enough time in the session to include nutritional activities, physical activities and enrichment every day. We would love to invite parents in to participate in food preparation with their children but there is not enough time in the session to do this” (P101-M).

Despite nutritional education being a mandatory element of the HAF guidance as stipulated by the DfE, 18 club leads acknowledged that delivering nutritional education was not a key priority at their HAF club, as “the providing of food to children is more a focus for us than the nutritional education” (P198-O). A lack of training was associated with this perception: “we do not deliver nutritional education as we are not trained to” (P164-O). Moreover, the importance of food provision often outweighed the nutritional education component of the programme: “providing food and snacks where applicable is important – teaching and discussing nutritional education is not at the top of the list” (P46-S). Limited guidance and lack of support influenced club leads’ decisions to not deliver nutritional education but acknowledged that “this is not what we do, however it does not mean to say we would not with the correct support” (P108-P) and one club lead delivering provision for children with disabilities noted that they “do provide nutritional education however it is limited due to [the children’s] needs and levels of understanding” (P181-O) and explained that they “have yet to come across any guidance from HAF that is sympathetic to the individual needs of children in our provision, which is disappointing.” (P181-O).

### 3.3 Perceived impacts of nutritional education

To compute the percentage of HAF club leads who did not perceive an improvement to each respective outcome, data were collapsed across responses of ‘not at all’ and ‘not much.’ By contrast, data were collapsed across responses of ‘somewhat’ and ‘very much’ to compute the percentage of participants who did perceive an improvement.

For each outcome for children, a greater percentage of HAF club leads perceived an improvement associated with nutritional education than the percentage of club leads who did not. Indeed, nutritional education was perceived to improve children’s willingness to try new foods (76.3%), diet (72%) and cooking confidence and competence (58.8%). Outcomes relating to knowledge and understanding were also perceived to improve, but to a lesser extent than the outcomes relating to observable skills and behaviours (Table 5).

**Table 5:** Frequency table showing perceived impacts of nutritional education for children (*n =* 118).

	*N*	%
Cooking confidence and competence		
N/A	20	16.95
Not at all	6	5.08
Not much	6	5.08
Neutral	17	14.41
Somewhat	37	31.36
Very much	32	27.12
Total	118	100
Diet		
N/A	7	5.93
Not at all	1	0.85
Not much	12	10.17
Neutral	13	11.02
Somewhat	55	46.61
Very much	30	25.42
Total	118	100
Willingness to try new foods		
N/A	6	5.08
Not at all	0	0.00
Not much	7	5.93
Neutral	15	12.71
Somewhat	36	30.51
Very much	54	45.76
Total	118	100
Understanding of food sustainability		
N/A	10	8.47
Not at all	10	8.47
Not much	14	11.87
Neutral	26	22.03
Somewhat	39	33.05
Very much	19	16.10
Total	118	100
Understanding environmental impacts and the food system		
N/A	10	8.47
Not at all	10	8.47
Not much	15	12.71
Neutral	33	27.97
Somewhat	34	28.81
Very much	16	13.56
Total	118	100
Understanding food provenance		
N/A	10	8.47
Not at all	12	10.17
Not much	13	11.02
Neutral	29	24.58
Somewhat	38	32.20
Very much	16	13.56
Total	118	100
Other		
N/A	64	54.24
Not at all	11	9.32
Not much	2	1.69
Neutral	27	22.88
Somewhat	6	5.08
Very much	8	6.78
Total	118	100

Collapsing the data in the same way as for children, a greater percentage of club leads perceived nutritional education to improve parents’/carers’ diets (38%), willingness to try new foods (34.5%) and budgeting (31%) than the percentage of club leads who did not. In addition, whilst 25% of club leads perceived nutritional education to improve parents’/carers’ cooking confidence and competence, a further 25% did not (Table 6). However, for the knowledge-based outcomes of (1) understanding of sustainability, (2) understanding of environmental impacts and the food system, and (3) understanding of food provenance, a greater percentage of HAF club leads did not perceive an improvement in each respective outcome compared to those who did (Table 6).

**Table 6:** Frequency table showing perceived impacts of nutritional education for parents/carers (*n =* 116).

	*N*	%
Cooking confidence and competence		
N/A	39	33.62
Not at all	19	16.38
Not much	10	8.62
Neutral	19	16.38
Somewhat	18	15.52
Very much	11	9.48
Total	116	100
Diet		
N/A	30	25.86
Not at all	15	12.93
Not much	7	6.03
Neutral	19	16.38
Somewhat	32	27.59
Very much	13	11.21
Total	116	100
Willingness to try new foods		
N/A	32	27.59
Not at all	16	13.79
Not much	9	7.75
Neutral	19	16.38
Somewhat	22	18.97
Very much	18	15.52
Total	116	100
Understanding of food sustainability		
N/A	33	28.45
Not at all	20	17.24
Not much	11	9.48
Neutral	25	21.55
Somewhat	16	13.79
Very much	11	9.48
Total	116	100
Understanding environmental impacts and the food system		
N/A	33	28.45
Not at all	20	17.24
Not much	13	11.21
Neutral	23	19.83
Somewhat	21	18.10
Very much	6	5.17
Total	116	100
Understanding of food provenance		
N/A	32	27.59
Not at all	22	18.97
Not much	9	7.75
Neutral	29	25.00
Somewhat	17	14.66
Very much	7	6.03
Total	116	100
Budgeting		
N/A	31	26.72
Not at all	18	15.52
Not much	12	10.34
Neutral	19	16.38
Somewhat	21	18.10
Very much	15	12.93
Total	116	100
Other		
N/A	71	61.21
Not at all	19	16.38
Not much	2	1.72
Neutral	18	15.52
Somewhat	4	3.45
Very much	2	1.72
Total	116	100

### 3.4 Guidance, skills and experience required to deliver nutritional education

Most HAF club leads (84.4%) believed that their staff/volunteers had the necessary skills and experience to deliver nutritional education. All HAF club leads were asked how staff/volunteers’ skills and experience, within the context of HAF nutritional education, could be improved. Responses (*n =* 115) were categorised into (1) additional resources and facilities, (2) working with external partners and skilled staff, (3) training and support, (4) funding for development, and (5) specific time to spend on improving their skills and experience.

In total, 22 club leads believed that additional resources and facilities would be beneficial in this context. For clarity, these data are reported in sub-section 3.1 regarding venues and facilities.

Working with external partners and skilled staff (i.e., nutrition-based skills) was suggested by 11 club leads to potentially improve staff/volunteers’ skills and experience regarding nutritional education, such as receiving “more support from our food providers in linking with our young people and families” (P152-M) and being “staffed by qualified teacher status staff” (P21-S). Staff/volunteers could observe sessions delivered by experts, and families could receive content directly from food-related professionals. However, club leads recognised related barriers, such as that outsourcing can be expensive, explaining that “more outside experts delivering free food sessions [would be useful], but at the moment outside providers are expensive” (P28-O) and “this is an issue when being a charity and money is tight” (P178-O).

Additionally, 70 club leads felt that enhanced training would improve staff/volunteers’ skills and experience for nutritional education delivery, and discussed general training such as learning “how they can use nutrition to engage children and broaden their minds when it comes to food” (P20-S), as well as specific, recognised qualifications such as a “Food Hygiene Certification course” (P139-S) or “Level 1 qualifications in nutritional guidance” (P220-O). This is an important tension to note, considering that 84% of HAF club leads believed that their staff had the necessary skills and experience to deliver to nutritional education. One club lead, who operated from a school, explicitly stated that they do not provide cooking classes due to a lack of training, and explained “to provide cooking classes, our site leaders would require food hygiene training and training on how to teach children how to safely prepare food in a fun, child-appropriate way. It would be good for them to also have basic nutrition training” (P211-S). Where training was provided to HAF staff/volunteers, club leads perceived an improvement in related skills and knowledge: “Our staff have had basic food hygiene training. This is really useful and gives a clear understanding of safer food practices” (P78-S).

To further improve staff/volunteers’ skills and experience, receipt of “funding for further training on nutrition and working with children” (P219-O) was suggested as beneficial by three HAF club leads, who explained that a designated allowance to support staff training would be valuable: “[It would be beneficial] if in the funding provided it allowed for us to have 2 days training as a team and allowed for preparation each day” (P19-S). Additionally, four club leads acknowledged that “if we had more time between confirming the commissioning of our work and the delivery, we would be able to train volunteers in-house beforehand rather than on the job” (P12-CC) and that they “have access to training but are expected to do this in our own time. This can be difficult as the staff/volunteers work in other jobs, making it extremely difficult to fit in travel and attend training, even if it is online” (P223-CC). Moreover, one participant who led provision operating from an adventure playground acknowledged that staff “are fully occupied with preparing, cooking and serving food” (P229-O), so additional time would allow for the development of skills and experience.

The remaining five HAF club leads were satisfied with the skills and experience of their staff/volunteers to deliver nutritional education, highlighting that their “staff are well trained” (P206-O) and that they “work in food tech as part of the national curriculum lessons and have food hygiene certificates” (P176-S).

Participants were also asked whether they felt the DfE guidance for HAF nutritional education was sufficient. Whilst 62.6% of club leads felt that the DfE guidance for HAF nutritional education was ‘just right,’ a further 28.7% of club leads believed that the guidance was ‘too little,’ with 8.7% feeling the guidance from the DfE was ‘too much.’ Participants were subsequently asked how much they relied upon the DfE guidance for HAF nutritional education. Collapsing the data across ‘not at all’ and ‘not much’ found that 33.3% of club leads did not rely upon the DfE HAF guidance for nutritional education. Collapsing the data across ‘somewhat’ and ‘very much’ found that 34.2% of club leads did rely on the guidance. However, only 5.3% of responding club leads relied upon the guidance ‘very much’ (Table 7).

**Table 7:** Frequency table displaying HAF club leads’ responses regarding how much they relied upon the DfE guidance for nutritional education (*n =* 114).

	*N*	%
Not at all	11	9.65
Not much	27	23.68
Neutral	37	32.46
Somewhat	33	28.95
Very much	6	5.26
Total	114	100

For further context, club leads were asked why they relied upon the DfE HAF guidance for nutritional education to their chosen extent. Responses (*n =* 77) were categorised into: (1) guidance is too limited, (2) already skilled or utilise guidance from elsewhere, (3) not aware of the guidance, (4) confusion surrounding what the guidance is, and (5) guidance is relied upon to support structure and content.

In total, 13 club leads noted that the guidance is too limited, explaining that they “do not receive much so do not rely on it” (P126-S) and that “not enough user-friendly information is available” (P216-O). Others noted that the guidance is too broad, explaining that “it is not very relevant to the needs of the children we work with” (P181-O) and “it never takes into account the socio-economic factors relevant to our community” (P21-S). Indeed, one participant stated that the guidance “is generic by nature so we add the context of the club we run to ensure the programme is fit for purpose and not just a tick box exercise” (P10-P) and others developed their own resources and guidance instead: “we have taken resources/information from other places which are more family friendly and especially worded more towards young people” (P220-O). To improve consistency across clubs, one respondent suggested that “it would be good for everyone taking part in the HAF programme to receive a pack of nutritional activities that you could run with groups, such as for different age groups and access to equipment, so that all groups have access to the same quality of resources” (P37-M).

Others (*n =* 27) noted that they do not rely upon the DfE guidance because they are already skilled in nutritional education or utilise guidance from elsewhere. For instance, one club lead said they “have been delivering these sessions for the past 7 years, [so] we have established our own standards” (P12-CC) and another explained that “I use and adapt my recipes from my experience of being a food teacher” (P29-S). Another highlighted concern surrounding the guidance being outdated, thus they relied upon external resources when developing their content: “In our club we have a manager with a diet and nutrition qualification, and we also have links with the Global Lead for Metabolic Health for Nestlé who is at the cutting edge of research into nutrition. The DfE is probably about a million years behind in its guidance” (P68).

Furthermore, eight club leads explained that they do not rely upon the DfE guidance for nutritional education because it is not suitable if they “only deliver activity and provide food” (P165-O) or “do not offer this service” (P34-LC). Moreover, two club leads stated that they “do not have the capacity to use the available advice and guidance” (P229-O) and that “there is too much work to do and reading advice and guidance from the DfE is another task” (P46-S). In addition, four club leads explained that they do not follow the guidance as they are “not really aware of it” (P108-P). Indeed, one of these club leads said that “this is the first we have heard of it” (P51-S), and another said that they are “yet to receive any information about DfE nutritional education” (P76-O).

Concerningly, when discussing why they did use DfE guidance for nutritional education, 11 club leads referred to guidance surrounding nutritional standards and content, such as considering allergies and School Food Standards, rather than the guidance relating to nutritional education (i.e., food-related educational activities). Whilst this may be considered an extension of food literacy or culinary nutrition, it does suggest some confusion regarding what the nutritional education guidance for HAF includes. For example, some HAF club leads discussed that their club had “very clear policies and procedures in place to ensure that we provide healthy snacks” (P154-O), and that the guidance was referred to when they “had a conversation during one particular evaluation and needed confirmation to look at Natasha’s Law” (P223-CC) which focused upon the guidance regarding nutritional contents of foods, rather than nutritional education. Likewise, others noted that they “have to rely on the guidance due to being expected to deliver in line with School Food Standards” (P48-S) and that they “adhere daily to the School Food Standards, plus we have highly motivated skilled chefs who know what nutrition is and how to make the most out of a few ingredients” (P19-S). Another respondent explained “we do not rely on it as we do not have the facility to prepare food on the premises” (P214-O), demonstrating further confusion surrounding the guidance which explicitly states that nutritional education does not have to involve food preparation activities.

Nevertheless, 14 club leads highlighted that “the guidance is used to structure the delivered sessions and to ensure we cover key nutritional information” (P41-LC), particularly where clubs are operated from organisations where food and nutrition are not their specialism: “because we are a creative arts organisation, we use the information a lot to guide us with our planning” (P152-M), and “as nutrition is not our main line of work, we check in with the guidance to ensure that we are delivering nutritional education to a good standard” (P134-O).

## 4 Discussion

The aim of this study was to explore the delivery, content, dose and perceived impacts of nutritional education, at the HAF club level, across England. Whilst most club leads were satisfied with the skills and experience held by staff and volunteers, the findings clearly show that nutritional education within HAF clubs was delivered in a variety of ways.

Most clubs operated from community venues and spaces (52%) and approximately two fifths (39%) operated from schools. Generally, however, the proportion of schools delivering HAF remains low, relative to community or voluntary organisations (16, 28), which may be related to a variety of factors including staff being on leave, school maintenance during holiday periods, lack of insurance cover or kitchen facilities being run by outside caterers. Moreover, whilst prior literature highlights schools as logistically suitable spaces for the delivery of nutritional education in holiday clubs, as they typically hold high-quality facilities and resources used during term-time for school catering and food education (29, 30), school kitchens are often inaccessible during the school holidays, even with a recent investment of £57 M from the DfE’s opening schools facilities fund (31); a common barrier similarly noted in prior literature regarding HAF nutritional education (26). Furthermore, clubs operating from parks and sports settings subsequently lacked cooking facilities, which hindered their ability to provide hot meals and deliver practical nutritional education activities. This is important in terms of related policy, as limited access to suitable kitchen facilities and equipment can restrict the type and quality of nutritional education that can be offered (26).

The DfE advocates for an assets-based and place-based approach to HAF, thus allowing flexibility for a variety of nutritional education activities to be delivered based on local material assets and need. As such, providers do not necessarily need access to cooking facilities and kitchen amenities to deliver nutritional education which meets DfE requirements. Rather, activities such as taste-tests, growing food or discussing nutrition constitute the broad range of activities that fall under nutritional education (13). Kitchen facilities and cooking equipment are, however, required for hands-on, practical food-related experiences, which are associated with significant improvement in food-related skills, knowledge and behaviours (20–22).

Some club leads reported that their HAF club lacked suitable facilities or space to deliver nutritional education. Thus, additional access to kitchens, cooking equipment and gardening space, particularly which children could use, as well as access to educational resources (e.g., recipe cards, nutritional information, worksheet booklets, information in other languages) were identified as resources that could improve clubs’ nutritional education offer at the local level. Although most participants felt their staff had suitable skills and experience to deliver nutritional education, training to upskill the workforce and recruiting food-related professionals were suggested as ways to improve clubs’ nutritional education provision; aligning with prior research demonstrating that nutritional education is often more effective when organised or led by an experienced food-related professional (22, 32). However, implementing such suggestions may prove difficult due to time and funding limitations, with prior research demonstrating that these barriers can subsequently reduce holiday club capacity and create difficulties sustaining provision (12, 14).

Discussing food and nutrition was the most common way of delivering, both to children and to parents/carers, which may align with the findings regarding clubs lacking access to relevant facilities to deliver experiential, practical food-related activities; as well as limited opportunities to outsource culinary professionals to deliver high-quality, practical content. Whilst some food preparation can be undertaken within HAF clubs operating from community settings (i.e., faith-based organisations, parks) where cooking facilities, such as ovens and hobs, are typically not required, the survey data suggests that some club leads struggle to deliver practical nutritional education overall. Indeed, most clubs spent less than 2 h per week discussing food and nutrition, with 30% of clubs not delivering this activity for children and 68% of clubs not delivering this activity to parents/carers. Likewise, practical food activities (e.g., food preparation, assembling, and cooking) were delivered by only half of HAF clubs (52%) for more than 2 h per week for children, and only 13% of HAF clubs for more than 2 h per week for parents/carers, with some clubs failing to deliver practical food-related activities entirely. Based upon experiential learning theory (33), practical food-related activities allow participants to develop an initial interest with food that can be continually developed (22). Repeated food exposure is key in such interventions and is associated with improved habitual consumption (34); children typically require 10–15 repeated exposures of foods before any significant food-related behaviour change is notable (35–37) and repeated teachings can foster deeper levels of learning and engagement through multiple opportunities for exposure to, and engagement with, the subject matter (38–40). As clubs often used a mixed mode of nutritional education delivery, incorporating a variety of activities to constitute this component of the programme, the limited amount of time spent on practical experiences such as food preparation and cooking may not be sufficient to drive changes in knowledge and behaviours at the individual level. It is important to note, however, that the HAF programme incorporates multiple mandatory elements alongside nutritional education (i.e., physical activities, cultural activities, health and wellbeing information), and such challenges may therefore relate to the time HAF leads have available to deliver all mandatory components each day.

Club leads perceived HAF nutritional education to improve levels of cooking confidence and competence, willingness to try new foods and diet, both for children and parents/carers. However, this perception does not correspond with how nutritional education was reported to be delivered in practice; although it is unclear whether club leads responded to the questions regarding the perceived impacts of nutritional education regarding the delivery at their specific club, or about nutritional education in general. Furthermore, a greater percentage of HAF club leads perceived observable skills (i.e., cooking confidence and competence, diet, willingness to try new foods) to be improved by nutritional education than knowledge-based outcomes (i.e., understanding of food provenance, environmental impacts, the food system and sustainability). As changes in skills and behaviours can be observed, and there are no formal assessment measures associated with HAF nutritional education, a lack of feedback surrounding knowledge acquisition may be associated with this finding. Given that no published studies have yet measured the impacts of HAF nutritional education on related outcomes, future quantitative research is required.

Fewer HAF clubs delivered nutritional education for parents/carers (46%) than for children (85%). Whilst this finding aligns with the DfE HAF guidance which stipulates that clubs should deliver daily nutritional education activities for children and weekly sessions for parents/carers, it seems a whole-family approach is rarely adopted; opposing findings from prior literature, and the updated DfE guidance, which advocates for clubs to involve the whole-family in nutritional education activities (13, 26). Including parents/carers in food-related interventions is often instrumental to skills and knowledge being utilised outside of the sessions (41–43). Even within the current study, the importance of involving the household budget holder and decision maker into HAF sessions, to improve the likelihood of skills and knowledge being translated into the family home, was recognised (42). However, stigma is sometimes associated with skills-based sessions for parents which can impact their attendance and engagement (26), and families facing food insecurity often hold sufficient nutritional skills and budgeting knowledge, but structural issues such as low income and the accessibility or affordability of required resources (i.e., ingredients, gas, and electricity) can hinder such skills and knowledge being utilised. Speculatively, this may explain why some clubs were reluctant to utilise a whole-family approach for nutritional education.

It is concerning that 15% of HAF clubs did not deliver any nutritional education activities for children, and 54% of clubs did not deliver any nutritional education activities for parents/carers; despite this being a mandatory component of the DfE HAF guidance. For some, nutritional education was not a priority, and club leads related its omission to a lack of material resources, training and funding, or focusing HAF resources on other areas of the programme (i.e., food provision). Recent HAF evaluations similarly note that clubs often fail to adhere to the DfE guidance for nutritional education delivery; indeed, only 33% of HAF clubs delivered nutritional education each day, with 1 in 10 providers not delivering any nutritional education at all (16). These findings reflect those highlighted by Round et al. (26) regarding equity of provision where nutritional education is delivered to various standards, if at all, across the HAF programme.

Such variation may relate to interpretation of the DfE HAF guidance, which has been highlighted as vague and ambiguous (26, 44). Only 5% of club leads within the current study reported that they very much relied upon the DfE guidance when planning, implementing and delivering the nutritional education component of HAF, with some clubs instead using information from elsewhere (e.g., Eatwell Guide, NHS, and National Curriculum). Ambiguity of guidance and vagueness surrounding expectation is related to poor communication and is a recognised barrier to effective working, underpinned by the ambiguity-conflict model (45). Whilst differences across HAF clubs most likely reflect place-based agendas, built upon available local assets (14), the vagueness and individual interpretation of the DfE guidance may also be associated with this variation in nutritional education delivery. Indeed, some club leads justified the omission of nutritional education by referring to the vagueness of the DfE guidance. Whilst it is therefore recommended that more precise guidance and enhanced clarity should be included in the DfE quality standards framework for HAF, changes to guidance should be approached with caution to ensure the guidance does not become too stringent which can reduce viability (19). A careful balance is required between recommending the most effective type of delivery (e.g., content and dose) in terms of driving behaviour change, whilst considering the local material assets available to deliver such activities. Moreover, some club leads demonstrated confusion between the guidance for nutritional education and other core components of HAF related to nutrition (i.e., School Food Standards, allergy information) and others did not follow the guidance for nutritional education as they were not aware of it. Whilst these elements may be considered an extension of food literacy or culinary nutrition, the DfE guidance for HAF includes separate guidance on the nutritional education component of HAF to the guidance for the food that is provided (i.e., School Food Standards, allergies). This raises concerns in terms of (1) whether all clubs receive guidance in advance of delivery, (2) whether they understand and use the guidance for the relevant core elements of the programme (e.g., health and safety), (3) whether the guidance is being passed on to those delivering at club level, and (4) whether clubs are provided with sufficient levels of funding to improve their delivery of hands-on cooking experiences.

### 4.1 Limitations of the study and further research

Whilst this study provides novel findings regarding how nutritional education is delivered within HAF clubs, methodological limitations should be recognised. Whilst disseminating the survey through the HAF Alliance and APPG on School Food was necessary as no sampling framework was available from the DfE, and although this sampling method has been successfully utilised in similar prior research regarding holiday provision (8), the sample remains relatively small (*n =* 147) and is not nationally representative of all HAF clubs. Thus, the findings from this study cannot be generalised, which is a caveat often recognised within survey research using non-probability purposive sampling (8). Nevertheless, the findings of this study provide a novel account of the current level of nutritional education delivered by clubs as part of the HAF programme. We recommend that future research should explore potential associations between HAF nutritional education and a range of outcome measures relating to health, wellbeing and knowledge. Informed by the literature in related areas such as culinary nutrition and food literacy (46, 47), as well as emerging research regarding the nutritional education component of HAF (26), we recommend that monitoring of this specific element of HAF be incorporated into an improved DfE HAF monitoring and evaluation framework. To support future research studies, we also recommend that the DfE align individual HAF pupil data with the National Pupil Database. This would enable researchers to explore the associations between HAF and school attendance and educational attainment.

### 4.2 Conclusion

This study makes a novel contribution to knowledge regarding the delivery of the nutritional education component of HAF. Whilst the DfE allows for local assets to be used, and flexibility in delivery to meet local community needs, the vagueness and ambiguity of the DfE guidance for nutritional education, alongside barriers including limited time and resources, prove challenging to the delivery of high-quality and equitable provision. Importantly, despite being a mandatory HAF requirement as stipulated by the DfE, 15% and 54% of clubs did not deliver any nutritional education for children or parents/carers, respectively. Further research is therefore required to explore the implementation, delivery and impact of nutritional education within HAF.

### 4.3 Policy recommendations

Whilst an assets-based and place-based approach supports local decision making based on local assets and need, HAF clubs operate from a wide range of venues (i.e., schools, community centres, parks, museums, an adapted double decker bus). It is clear that not all HAF clubs have access to the required facilities and/or space to deliver practical food-related activities, which are most effective in driving changes in skills, knowledge and behaviours (21, 22). Moreover, given that the recently updated DfE HAF guidance now includes a subsection titled ‘ensuring providers meet the programme standards’ (13), it is recommended that the DfE further develops its governance, quality assurance and monitoring framework for local authorities and HAF clubs; providing more detailed guidance on all elements of HAF (e.g., delivering nutritional education sessions) alongside examples of good practice and additional learning resources that may include online training, materials, etc. It is recommended that age-appropriate nutrition and food activities are developed in accordance with the national curriculum, which may be beneficial in driving long-term change in food-related skills, knowledge, attitudes and behaviours.

In addition, it is recommended that the DfE uses research evidence to inform providers of the importance of nutritional education for children and families, and provide research and evidence-based examples of best practice. Furthermore, some club leads noted that a lack of training regarding nutritional education limited their offer. Hence, it is also recommended that the DfE allocate specific top-up funding for providers to access training opportunities to upskill the workforce.

A more coherent approach to working in partnership with schools could provide further opportunities for HAF clubs to provide attendees with food growing and harvesting projects that could prove beneficial to schools, HAF clubs and attendees. Alternatively, there may be opportunity for HAF clubs to link to community gardens and allotments.

Whilst prior literature notes the importance of adopting a whole-family approach to nutritional education, as parents are typically the household budget holders and decision makers (26, 42), the findings from this study highlight that many HAF clubs do not deliver nutritional education specifically to parents/carers, nor offer sessions that involve the whole family. It is therefore recommended that the DfE include an evidence-based rationale for the importance of utilising a non-stigmatising, whole-family approach to nutritional education to improve the likelihood of skills and knowledge being transferred and embedded into the home.

It is, however, important to note that any amendments to HAF guidance should be considered with caution to ensure it does not become too strict, which could otherwise reduce viability of HAF delivery at the local level (19). Development and implementation should be conducted through co-production methods with the relevant HAF stakeholders.

## Data Availability

The raw data supporting the conclusions of this article will be made available by the authors, without undue reservation.
